# Relaxation music for stress alleviation: Exploring relationships of audio features and listener profiles

**DOI:** 10.1177/10298649261421169

**Published:** 2026-03-11

**Authors:** Catherine Tan, Anastasios Mavrolampados, Alessandro Ansani, Marianne Taipale, Friederike Koehler, William M. Randall, Suvi Saarikallio

**Affiliations:** 1 Centre of Excellence in Music, Mind, Body and Brain, University of Jyväskylä, Finland

**Keywords:** relaxation, stress alleviation, music listening, anxiety, depression, music selection

## Abstract

Stress is an increasingly prevalent state experienced by many individuals, but mood regulation and relaxation can alleviate some of its associated symptoms. While music listening has generally been shown to support stress alleviation, less is known about how individual differences potentially guide the choice of music for this purpose. The present study focused on depression and anxiety as individual differences that might relate to the selection of relaxation music. Through an online questionnaire, 93 participants provided ~20 minutes (median number of tracks per participant = 6) of self-selected relaxation music and completed questionnaires measuring depression, anxiety and previous use of music for mood regulation and adaptive functions. We compared Spotify audio features of the relaxation music to general music and found that relaxation music had higher levels of Acousticness and lower levels of Speechiness, Energy, Valence, Liveness, Instrumentalness, Danceability and Loudness. Through clustering, we identified three subgroups of relaxation music (i.e., ‘loud-energetic-danceable’, ‘neutral-acoustic’ and ‘mellow-instrumental’). Finally, results showed that higher depression scores increased the likelihood of selecting relaxation music from the ‘loud-energetic-danceable’ cluster over the remaining two, while higher anxiety scores increased the likelihood of selecting music from the ‘mellow-instrumental’ cluster over the ‘loud-energetic-danceable’ cluster. Previous use of music for mood regulation, but not adaptive functions, also predicted music selection for relaxation. This research advances our understanding of how different individuals use music to reduce stress and relax and provides guidance on how music can adaptively cater for these purposes.

## Stress and the need for relaxation in daily life

Navigating everyday life in the modern world offers endless opportunities but also presents challenges that test the elasticity of human resilience. When balancing tasks becomes too difficult, it can lead to the experience of stress, ‘a state of worry or mental tension’ that can be associated with physiological and emotional changes such as headaches, body pains, sleep difficulties and feelings of anxiety, depression, or irritability (World Health Organization, 2023a, 2023b). Recent research has noted that experiences of psychological and emotional distress, deriving from factors like stress, have been increasing globally (Blanchflower & Oswald, 2020; Daly & Macchia, 2023; Keyes & Platt, 2024; Zhang et al., 2022). Though a universal response that is essential to our adaptability and survival, individuals experience and manage stress impediments differently, leading to various wellbeing and performance outcomes.

One way to alleviate stress is through regulating mood and enhancing relaxation. Smith (2007) suggested that relaxation can be conceptualised and achieved through basic relaxation, core mindfulness, positive energy and transcendence, which can further be subdivided into 12 different states varying from sleepy to energetic in terms of their arousal level. Taken together, research towards accessible relaxation interventions that embrace the multiple forms of relaxation needs to be considered and taken proactively to foster healthier societies.

## Music as a tool for stress alleviation and relaxation

A growing amount of evidence on diverse populations and contexts has indicated that music interventions (e.g., singing, instrument playing, music listening and improvisation) can support stress alleviation and related down-regulation processes like relaxation or calming (Barlow, 2004; de Witte et al., 2020; Finn & Fancourt, 2018). In hospital and clinical settings, music interventions have helped patients cope with stress and anxiety stemming from their treatments and recovery (Bradt et al., 2015; Iyendo, 2016; Karadag et al., 2019) and for healthcare workers, music has been found to support the management of work-related stressors (Colin et al., 2023). The adoption of music listening as a self-regulatory tool for mood regulation, stress management and relaxation, is also apparent in the everyday lives of the general population (Adiasto et al., 2023; Baltazar et al., 2019; Boer & Fischer, 2012; Randall & Rickard, 2017; Saarikallio & Erkkilä, 2007; Schäfer et al., 2013). Specifically for cases of stress coping, Krause et al. (2023) found that individuals turn to music listening to regulate a range of stressors, including those pertaining to social, financial, work and personal responsibilities.

## Influences of individual differences in mental health on the use of music

While practically anyone can use music listening for mood regulation and relaxation, research has revealed that certain populations are more susceptible to do so. For example, it has been found that individuals who experienced higher levels of stress or neuroticism listened more to music for emotional purposes (Chamorro-Premuzic & Furnham, 2007; Getz et al., 2014; Miranda et al., 2010). In such cases, music can facilitate mood regulation strategies (B-MMR; Saarikallio, 2012; Saarikallio & Erkkilä, 2007) or serve coping functions (AFML; Groarke & Hogan, 2018), but individual differences in mental health may influence whether these lead to adaptive or maladaptive outcomes (Cheong-Clinch & McFerran, 2016; Garrido & Schubert, 2015; Morgan & Marroquín, 2024). In a study by ter Bogt et al. (2017), individuals with higher levels of anxiety and depression frequently found music to adaptively support their management of daily stressors and sorrows through offering consolidation. However, for youth with depression, music was found to regulate mood in both healthy and unhealthy ways (Miranda & Claes, 2009; Saarikallio et al., 2015).

Beyond regulation strategies, mental health has also been found to shape music preferences, which may in turn influence the music used for mood regulation. Research has displayed a reciprocal relationship between depression and musical preferences, wherein, clinically depressed individuals demonstrated preferences for certain musical genres (Punkanen et al., 2011), and inversely, adolescents with a narrower range of music preferences tended to experience more developmental difficulties (Schwartz & Fouts, 2003).

## The role of musical content for relaxation

Though individual differences may contribute to music engagement for stress alleviation, other research approaches have focused on the role of music features in emotion perception and induction, two processes involved in musical mood regulation and relaxation. For the former, studies have relied on participant evaluations of music features (e.g., tempo, mode, pitch, harmony and rhythm) to understand how they communicate emotions through music (Gabrielsson & Lindström, 2010; Hevner, 1936, 1937; Juslin & Laukka, 2004); whereas, the latter attempts to understand how music features activate psychophysiological mechanisms to induce emotion (e.g., BRECVEMA framework; Juslin & Västfjäll, 2008). When it comes to relaxation music in particular, slow and soft instrumental music with low rhythmic, harmonic and melodic complexity has been most perceived as relaxing and calming across expert (i.e., musicians and music therapists), commercial and general populations (Gabrielsson & Lindström, 2010; Tan et al., 2012; Wolfe et al., 2002). Thus, clinical practitioners have focused on these musical characteristics as frameworks to inform music selection for stress alleviation interventions (de Witte et al., 2020, 2022; Tan et al., 2012), but do these reflect in self-guided musical relaxation in everyday life?

Recent studies have utilised Spotify’s metadata and audio features (e.g., Valence, Danceability, Liveness) to characterise music for different functions (e.g., Duman et al., 2022 on dance; Scarratt et al., 2023a on sleep). Tan & Harrison (2025) used the same methodology to characterise self-selected music for different music mood regulation strategies while also considering how participant background variables predict the usage of the B-MMR strategies and their associated music. Only a few studies have adopted this method to characterise self-selected music for relaxation (e.g., Adiasto et al., 2023; Baltazar & Västfjäll, 2019); however, evaluations on how participant profiles (e.g., differences in demographics, usage of music mood regulation strategies, mental health) relate to music engagement and selection were not taken. By understanding relaxation music at an audio feature level in conjunction with the study of individual differences and musical relaxation strategies, the development of personalised cost-effective interventions for stress alleviation like evidence-based relaxation music playlists could be supported.

## Aim of the current study

The reviewed literature reveals that for musical relaxation to be successful, not only the music itself matters, but also the individuals behind selecting the music. Despite this, the interactions of these factors remain relatively unexplored. The aim of this study was to better understand the characteristics of self-selected music for relaxation and how they may relate to varying listener profiles, both on a mental health and musical level. Three research questions guided this investigation:

1. What are the characteristics of relaxation music as measured by Spotify audio features?
2. Can we identify clusters of relaxation music, each with different measures of Spotify audio features?
3. How do individual differences in mental health (i.e., depression and anxiety) and in musical emotion regulation (i.e., B-MMR and AFML) predict the selection of relaxation music?

Based on previous research, we expect relaxation music to generally be instrumental, slow and soft. As regards to how individual differences in mental health and musical mood regulation relate to music selection, we kept the investigation exploratory due to lack of prior research.

## Methods

### Participants

Ninety-three participants (female = 65, male = 23, other = 5) between 18 and 33 years old (*M*_age_ = 24.30, *SD* = 3.69) completed a questionnaire which was part of a stress alleviation experiment (Taipale et al., 2025). Since the study was conducted in Finnish, our sample consisted of native Finnish speakers or individuals with a proficient level of immersion in Finnish culture. Recruitment for the study was completed using social media ads (i.e., Facebook and Instagram) and mailing lists associated with the University of Jyväskylä’s Department of Music, Art and Culture. All protocols of the study were completed in accordance with the University of Jyväskylä’s ethics guidelines and the study was reviewed by the university’s Human Sciences Ethics Committee.

### Procedure

The current data were collected as part of a stress alleviation experiment (Taipale et al., 2025), for which the participants were asked to provide a playlist of their own relaxation music. Upon expressing interest in participating, the information sheet, consent form and online questionnaire were shared electronically to participants through the online survey platform Webropol. At this time, participants were informed of the overall study, including the stress induction and alleviation experiment that would take place in the laboratory. After written consent was provided, participants completed music and psychometric measures (see below) and were then asked to provide a list of self-chosen relaxation music totalling around 20 minutes in duration. For each song on their list, participants were requested to provide the track name and artist. The specific prompt (translated from Finnish to English) used for this task was: ‘For the listening experiment in the Musica building, we need a 20 minute list of music that you would like to use for relaxation. Write here the songs and performers you have chosen’.

### Measures

#### Anxiety and depression

The Finnish version of the Hospital Anxiety and Depression Scale (HADS; Aro et al., 2004; Zigmond & Snaith, 1983) was used to gather participants’ states of anxiety and depression. The scale is composed of 7 items for each subscale, anxiety and depression, and responses for each item are ranked on a 4-point scale. Scores for each subscale are totalled and can range between 0 and 21. Levels of high anxiety and depression are represented by higher scores.

#### Individual uses of music mood regulation strategies

To assess participants’ use of seven music mood regulation strategies (i.e. Entertainment, Revival, Strong Sensation, Diversion, Discharge, Mental Work and Solace), the Finnish version of the 21-item Brief Music in Mood Regulation scale (B-MMR; Saarikallio, 2012) was used. Participants indicated their agreement to each item on a 5-point Likert scale from ‘1 (Strongly disagree)’ to ‘5 (Strongly agree)’. The mean scores for each subscale and the collective B-MMR were calculated.

#### Adaptive functions of music listening

Four subscales of the Adaptive Functions of Music Listening scale (AFML; Groarke & Hogan, 2018) were used to collect participants’ functions of using music listening for wellbeing. Specifically, four items for Stress regulation, four items for Rumination, two items for Sleep and seven items for Anxiety regulation were used. Participants rated their agreement on each item using a 5-point Likert scale ranging from ‘1 (Strongly disagree)’ to ‘5 (Strongly agree)’, mean scores for each subscale were calculated. The items were translated from English to Finnish by two researchers.

### Datasets

The MUSICONNECT Relaxation Music Dataset was created using the music lists our participants provided. The initial dataset consisted of 642 tracks; however, tracks without artist details provided from participants (*n* = 4) and those without audio feature measures available from the Spotify API (*n* = 2) were excluded. Tracks that appeared in multiple participants’ lists (*n* = 34) were included only once in the dataset. The final dataset used for analyses consisted of 598 unique tracks.

To identify how the characteristics of the MUSICONNECT Relaxation Music Dataset differ from those of general music, the Music Streaming Sessions Dataset (MSSD; Brost et al., 2019) was used for comparison. This dataset is made up of 3 706 623 unique tracks.

### Audio features of analyses

To characterise the music in the datasets, we retrieved nine high-order features from the Spotify API (Spotify, n.d.) that were derived from the tracks’ audio signal and describe some key musical dimensions. A description of each feature is shown in Table 1.

**Table 1.** Spotify API audio features (Spotify, n.d.).

Feature	Description
Danceability	‘Danceability describes how suitable a track is for dancing based on a combination of musical elements including tempo, rhythm stability, beat strength, and overall regularity. A value of 0.0 is least danceable and 1.0 is most danceable’.
Energy	‘Energy is a measure from 0.0 to 1.0 and represents a perceptual measure of intensity and activity. Typically, energetic tracks feel fast, loud, and noisy. For example, death metal has high energy, while a Bach prelude scores low on the scale. Perceptual features contributing to this attribute include dynamic range, perceived loudness, timbre, onset rate, and general entropy’.
Loudness	‘The overall loudness of a track in decibels (dB). Loudness values are averaged across the entire track and are useful for comparing relative loudness of tracks. Loudness is the quality of a sound that is the primary psychological correlate of physical strength (amplitude). Values typically range between -60 and 0 db’.
Speechiness	‘Speechiness detects the presence of spoken words in a track. The more exclusively speech-like the recording (e.g. talk show, audio book, poetry), the closer to 1.0 the attribute value. Values above 0.66 describe tracks that are probably made entirely of spoken words. Values between 0.33 and 0.66 describe tracks that may contain both music and speech, either in sections or layered, including such cases as rap music. Values below 0.33 most likely represent music and other non-speech-like tracks’.
Acousticness	‘A confidence measure from 0.0 to 1.0 of whether the track is acoustic. 1.0 represents high confidence the track is acoustic’.
Instrumentalness	‘Predicts whether a track contains no vocals. ‘Ooh’ and ‘aah’ sounds are treated as instrumental in this context. Rap or spoken word tracks are clearly ‘vocal’. The closer the instrumentalness value is to 1.0, the greater likelihood the track contains no vocal content. Values above 0.5 are intended to represent instrumental tracks, but confidence is higher as the value approaches 1.0’.
Liveness	‘Detects the presence of an audience in the recording. Higher liveness values represent an increased probability that the track was performed live. A value above 0.8 provides strong likelihood that the track is live’.
Valence	‘A measure from 0.0 to 1.0 describing the musical positiveness conveyed by a track. Tracks with high valence sound more positive (e.g. happy, cheerful, euphoric), while tracks with low valence sound more negative (e.g. sad, depressed, angry)’.
Tempo	‘The overall estimated tempo of a track in beats per minute (BPM). In musical terminology, tempo is the speed or pace of a given piece and derives directly from the average beat duration’.

### Analyses

To complete the following data analyses and data visualisations, the R Studio software was used (R Core Team, 2023).

#### Audio features of relaxation music

Measures of audio features extracted from the Spotify API were used to analyse the characteristics of relaxation music in this study. To compare the audio feature measures of each music dataset while accounting for the unequal variances and dataset sizes, Welch’s *t*-tests were performed. The effect sizes of the differences between the audio features of the datasets were calculated using Cohen’s *d*. The values were interpreted as small (*d* = 0.2), medium (*d* = 0.5) and large (*d* = 0.8) (Cohen, 1988).

#### Clusters of relaxation music

To identify main feature patterns in relaxation music, k-means clustering analysis was performed on normalised data with Euclidean distances. We used the *nbClust* package (Charrad et al., 2025) to assess dimensionality and identify the optimal number of clusters. We simultaneously tested 24 different clustering indices (e.g., KL, Silhouette value, Beale index, *SD* index) and computed the optimal number of clusters suggested by each. Using majority rule, the results revealed 9 indices alluding to a three-cluster solution.

#### Predicting relaxation music selection from participant profiles

A Mixed Multinomial Logit model was used to model how participant profiles associated with the selection of music from the clusters of relaxation music. We used the *mclogit* package (Elff, 2022) to complete the modelling.

## Results

### Characterising relaxation music

**Figure 1.**
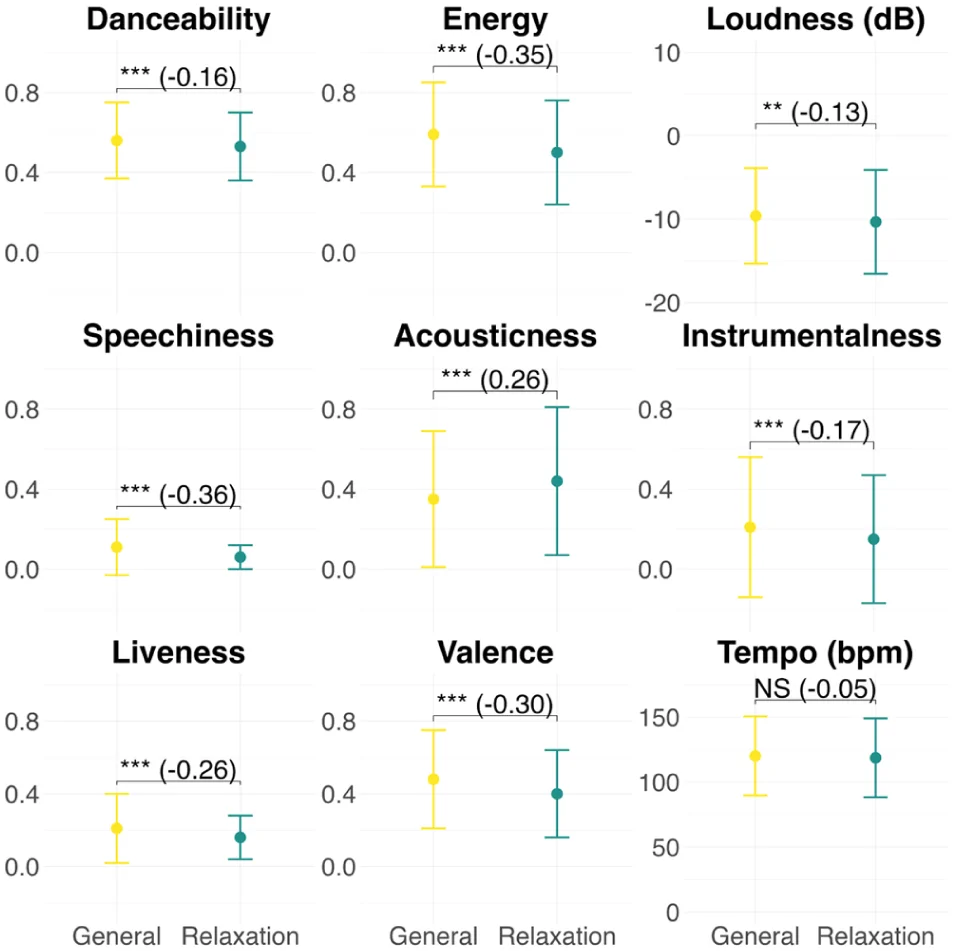
Audio feature comparison between general music and relaxation music. Each panel shows mean comparisons of an audio feature with error bars representing standard deviations. Asterisks represent *t*-test significance and Cohen’s *d* values are illustrated in the brackets. **, *p* < .01 and ***, *p* < .001.

Welch’s *t*-tests showed significant differences between Spotify audio features of the relaxation music dataset and the general music dataset at small effect sizes. Specifically, we observed that relaxation music had higher Acousticness (*p* < .001, *d* = 0.26) and lower Speechiness (*p* < .001, *d* = −0.36), Energy (*p* < .001, *d* = −0.35), Valence (*p* < .001, *d* = −0.30), Liveness (*p* < .001, *d* = −0.26), Instrumentalness (*p* < .001, *d* = −0.17), Danceability (*p* < .001, *d* = −0.16) and Loudness (*p* < .01, *d* = −0.13). The results are visualised in Figure 1 and a full overview can be found in Table 2.

**Table 2.** Results of Welch’s *t*-tests and Cohen’s *d* between the relaxation and general music datasets.

Spotify audio features	Relaxation dataset (*N* = 598)		General music dataset (*N* = 3 706 623)		Welch’s *t*-test		Cohen’s *d*
	Mean	*SD*	Mean	*SD*	*t*-value	*p*-value	*d*-value
Danceability	0.53	0.17	0.56	0.19	–4.31	*p* < .001	–0.16
Energy	0.50	0.26	0.59	0.26	–8.46	*p* < .001	–0.35
Loudness (dB)	–10.32	6.23	–9.60	5.73	–2.83	.005	–0.13
Speechiness	0.06	0.06	0.11	0.14	–20.37	*p* < .001	–0.36
Acousticness	0.44	0.37	0.35	0.34	5.95	*p* < .001	0.26
Instrumentalness	0.15	0.32	0.21	0.35	–4.58	*p* < .001	–0.17
Liveness	0.16	0.12	0.21	0.19	–10.19	*p* < .001	–0.26
Valence	0.40	0.24	0.48	0.27	–8.15	*p* < .001	–0.30
Tempo (BPM)	118.67	30.33	120.07	30.43	–1.13	.259	–0.05

### Clusters of relaxation music

**Figure 2.**
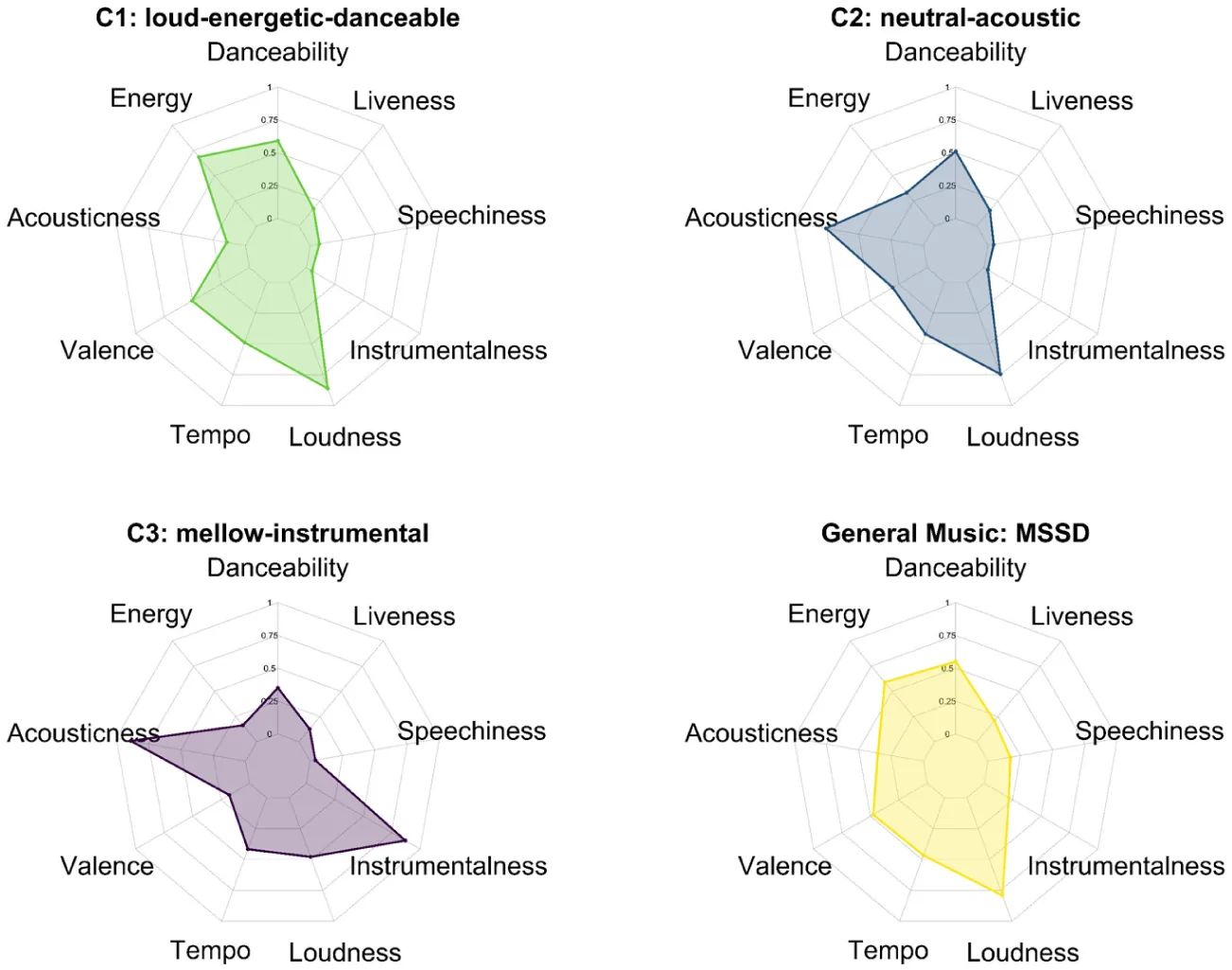
Radar plots of the nine audio features characterising Cluster 1 (‘loud-energetic-danceable’), Cluster 2 (‘neutral-acoustic’), Cluster 3 (‘mellow-instrumental’) and the general music dataset (MSSD).

K-means revealed three different clusters, each characterised by distinct patterns of Spotify audio features (Figure 2). The means and standard deviations of the audio features for each cluster are in Table 3. To provide an overview of the tracks in each cluster, we present the 10 closest tracks to each cluster centroid (in descending order) in Table 4.

**Table 3.** Means and standard deviations of audio features for the clusters of relaxation music.

Spotify audio features	Cluster 1 (*N* = 324)		Cluster 2 (*N* = 195)		Cluster 3 (*N* = 79)	
	Mean	*SD*	Mean	*SD*	Mean	*SD*
Danceability	0.59	0.15	0.51	0.15	0.35	0.16
Energy	0.69	0.16	0.33	0.16	0.16	0.18
Loudness	0.86	0.06	0.74	0.10	0.48	0.19
Speechiness	0.07	0.08	0.04	0.03	0.04	0.01
Acousticness	0.14	0.16	0.75	0.18	0.89	0.17
Instrumentalness	0.05	0.17	0.03	0.09	0.87	0.12
Liveness	0.17	0.12	0.16	0.11	0.13	0.09
Valence	0.50	0.24	0.30	0.16	0.18	0.17
Tempo	0.49	0.18	0.42	0.19	0.42	0.25

**Table 4.** Ten tracks closest to the centroid of each cluster.

Cluster	Song name	Artist name
1	Paperitähdet	Popeda
	The Phantom Of the Opera–From ‘The Phantom Of The Opera’ Motion Picture	Andrew Lloyd Webber
	Home	Depeche Mode
	Sininen ja valkoinen	Jukka Kuoppamäki
	Misty Morning, Albert Bridge	The Pogues
	Heaven	Bryan Adams
	My Sweet Lord (2014 Remaster)	George Harrison
	Sweet Dreams, TN	The Last Shadow Puppets
	ウィルオウィスプ	Kenshi Yonezu
	Toxicity	System Of A Down
2	Patience	Guns N’ Roses
	Rise Up	Andra Day
	Science	Niall Horan
	Lay All Your Love On Me	The Butterfly Effect
	Tulkoon mitä vaan (feat. Aili Järvelä ja Jutta Rahmel) [Vain elämää kausi 10]	Vesala
	Can You Feel the Love Tonight–End Title	Elton John
	Trip To Skye	John Whelan
	House of Cards	Alexander Stewart
	You Raise Me Up	Westlife
	Seigfried	Frank Ocean
3	Lights Are On–Instrumental	Edith Whiskers
	Evening Wind	Joe Hisaishi
	Weightless Part 4	Marconi Union
	Reprise	Joe Hisaishi
	River Flows In You	Yiruma
	Peperomia Seedling	Green-House
	Piano Concerto No. 2 in C Minor, Op. 18: 2. Adagio sostenuto	Sergei Rachmaninoff, London Symphony Orchestra
	Silence	TWO LANES
	Peace Piece	Green-House
	Parfum d’étoiles	Ichiko Aoba

Cluster 1, composed of 324 tracks, was characterised by high Loudness and Energy; moderate levels of Danceability, Valence and Tempo; and low levels of Liveness, Acousticness, Speechiness and Instrumentalness (e.g. *Sininen ja valkoinen* by Jukka Kuoppamäki, *Bad Habits* by Ed Sheeran, *Italia* by Isac Elliot). To move away from labelling the clusters by genre, of which can cause ambiguity, we choose to name our clusters by their audio features. Based on this cluster’s high scores in Loudness and Energy and moderate Danceability, Cluster 1 was named ‘loud-energetic-danceable’.

Cluster 2, composed of 195 tracks, was characterised by high Acousticness and Loudness; moderate levels of Danceability and Tempo; and low levels of Energy, Valence, Liveness, Speechiness and Instrumentalness (e.g., *Lover* by Taylor Swift, *Kahdestaan* by Ida Paul & Kalle Lindroth, *Ikävä* by emma & matilda). This cluster was named ‘neutral-acoustic’ after its high Acousticness and moderate-low Energy and Valence.

Cluster 3, composed of 79 tracks, was characterised by high Acousticness and Instrumentalness; moderate Loudness, Tempo and Danceability; and low levels of Valence, Energy, Liveness and Speechiness (e.g. *Piano Concerto No. 2 in C minor, Op 18: 2. Adagio sostenuto* by Sergei Rachmaninoff; *Time* by Hans Zimmer; *The Sixth Station* by Joe Hisaishi). With tracks that are more instrumental and with low levels of Valence and Energy, we named this cluster ‘mellow-instrumental’.

The defining audio features for each cluster are the same audio features that distinguish them from general music. Interestingly, Cluster 1 had higher mean measures of Loudness, Energy and Danceability than the general music dataset. Speechiness and Liveness shared similar measures across the three clusters, but were all consistently lower than the measures of general music.

### Predicting the likelihood of selecting relaxation tracks from a given cluster based on participants’ mental health and musical emotion regulation profiles

A Mixed Multinomial Logit model (MMNL; McFadden & Train, 2000) strategy was implemented to predict the odds^1^ of selecting tracks from the different clusters given individual differences.

Three models were trained using three different sets of predictors. In Model 1, the predictors were both facets of the HADS (i.e., depression and anxiety); in Model 2, the seven B-MMR strategies; finally, in Model 3, the predictors were the AFML subscales. Z-score transformations were performed on the predictors for the modelling.

Due to the repeated-measure nature of the data (i.e., each participant listed multiple tracks) and the fact that participants provided different numbers of tracks (ranging from 3 to 20), participants were modelled as random intercepts. We used the Penalised Quasi-Likelihood (PQL) method to handle the complexity of the random effects in the models and ensure robust estimates for the clustered data structure. The Maximum Likelihood estimator was chosen to evaluate the fixed effects. Prior to modelling, we checked for multicollinearity among the predictors by calculating Variance Inflation Factors (VIF) using multiple linear regression models. No concerning levels of multicollinearity were detected, so no predictors were dropped (VIF < 5; James et al., 2021).^2^

McFadden’s pseudo-*R*^2^ (ρ^2^) was computed, consistent with the multinomial nature of the model. All models showed a very good fit (ρ^2^_Mod1_ = 0.26; ρ^2^_Mod2_ = 0.27; ρ^2^_Mod3_ = 0.26) according to McFadden’s suggestion that ‘values of 0.2 to 0.4 for ρ^2^ represent an excellent fit’ (McFadden, 1979, p. 307). The percentages of pieces within each cluster predicted by the models correlated almost perfectly with the observed percentages (*r*_s Mod1_ > .93, *p* < .001; *r*_s Mod2_ > .91, *p* < .001; *r*_s Mod3_ > .93, *p* < .001).

The multiclass accuracy of each model was measured by computing the ratio of correct predictions and the total number of predictions. All models had high accuracy (Acc_Mod1_ = 74.96%; Acc_Mod2_ = 74.96%; Acc_Mod3_ = 75.11%, chance = 54%), namely, slightly more than 20 percentage points above the chance level.^3^ Finally, the models achieved an overall performance in terms of weighted accuracy of F1_weighted Mod1&2_ = 73.97% and F1_weighted Mod3_ = 74.24%, indicating a good classification performance while accounting for cluster imbalance.

#### HADS model

**Figure 3.**
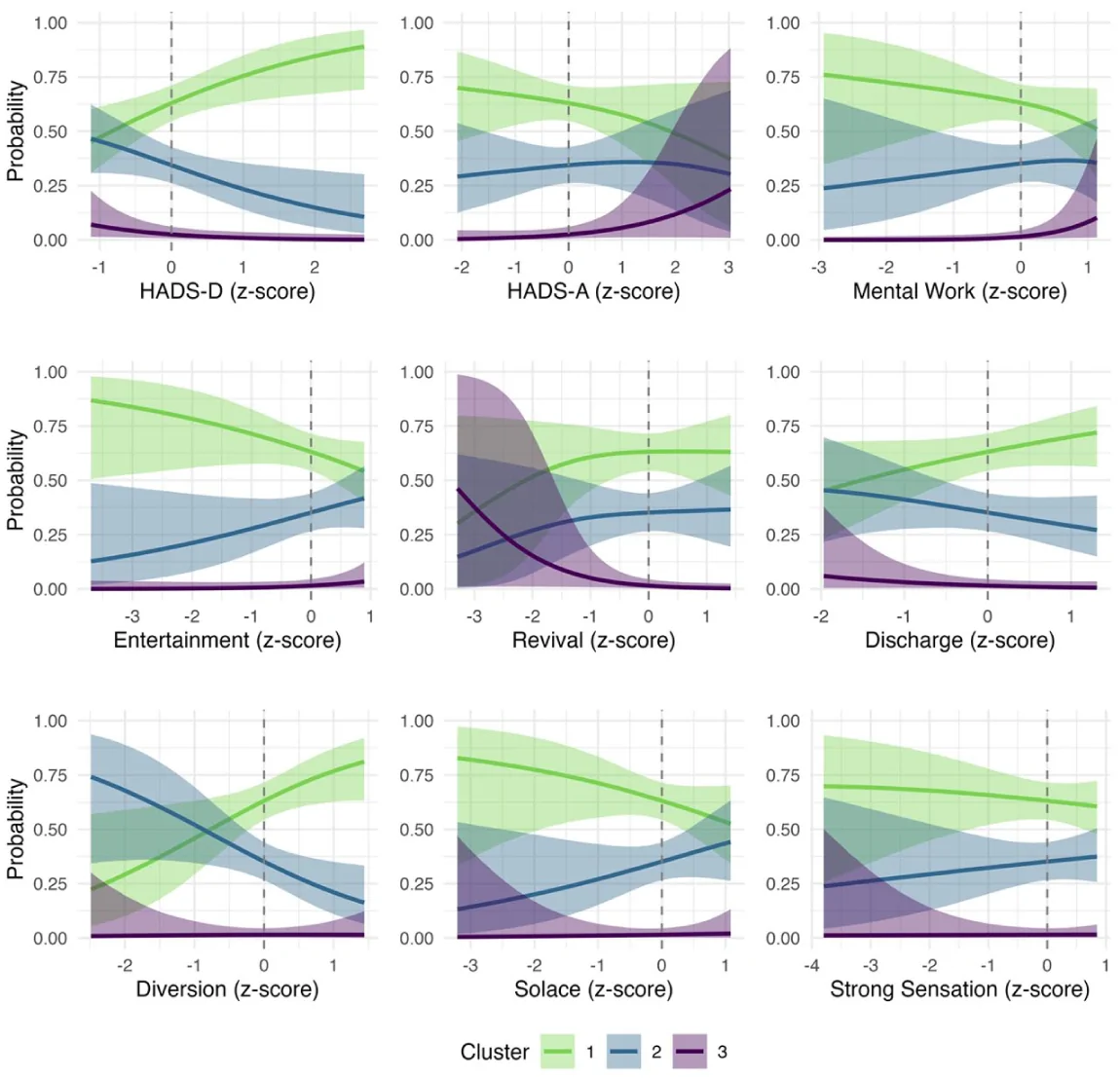
Probability of choosing a track from a particular cluster based on HADS and B-MMR scores. The mean trends and their 95% confidence intervals are depicted. The dashed line represents the mean value of each predictor.

HADS-D led to an increase in the probability of choosing pieces from Cluster 1 rather than 2 (*OR* = 1.65, 95%CI [1.05,2.59], *p* = .029) or 3 (*OR* = 2.25, 95%CI [1.25,4.03], *p* = .006). Conversely, HADS-A increased the odds of Cluster 3 rather than 1 (*OR* = 1.80, 95%CI [1.01,3.23], *p* = .006). In summary, this model suggests that individuals with higher depression are more likely to select ‘loud-energetic-danceable’ music to relax as opposed to the other two clusters, whereas individuals with higher anxiety are more likely to select ‘mellow-instrumental’ music as opposed to ‘loud-energetic-danceable’ music for the same purpose. The results of this model are visualised in Figure 3.

#### B-MMR model

For a 1SD increase in the B-MMR Mental Work strategy, the odds of choosing a piece from Cluster 3 became significantly higher than those of choosing a piece from Cluster 1 (OR = 3.12, 95% CI [1.26, 7.72], *p* = .013). The same increase in the B-MMR Entertainment strategy led to a higher relative likelihood of choosing a piece from Cluster 3 as opposed to Cluster 1 (OR = 1.83, 95% CI [0.99, 3.38], *p* = .051). Conversely, Revival (OR = 0.52, 95% CI [0.26, 1.00], *p* = .051) and Discharge (OR = 0.60, 95% CI [0.34, 1.03], *p* = .067) seem to have led to decreased odds of choosing pieces from Cluster 3 rather than 1. Lastly, Diversion decreased the odds of choosing pieces from Cluster 2 as opposed to Cluster 1 (OR = 0.55, 95% CI [0.32, 0.96], *p* = .037). To summarise, this model suggests that individuals who more frequently use the B-MMR strategies of Mental Work and Entertainment are more likely to select ‘mellow-instrumental’ music over ‘loud-energetic-danceable’ music for relaxation, while those who more frequently use Revival and Discharge display the opposite trend. The likelihood of selecting ‘neutral-acoustic’ music over ‘loud-energetic-danceable’ music might increase for individuals who use Diversion more frequently. The results of this model are also visualised in Figure 3.

#### AFML model

No significant effects were found in the AFML model.

## Discussion

Turning to music listening as a tool for stress alleviation and relaxation is becoming more common within the general population (Getz et al., 2014; Krause et al., 2023; Randall & Rickard, 2017; Schäfer et al., 2013). The current study investigated the characteristics and variability of self-selected music for relaxation. Moreover, we examined how mental health and musical emotion regulation profiles predicted music selection for this purpose.

### Characteristics of relaxation music

Through creating the MUSICONNECT Relaxation Music Dataset, we broadly summarise that self-selected relaxation music is typically softer and more acoustic than general music. These findings are largely in line with our hypothesis and reinforce previous suggestions that these features define the perception of relaxing music (Gabrielsson & Lindström, 2010; Tan et al., 2012; Wolfe et al., 2002). Utilising the Spotify audio features, our analysis revealed that relaxation music, at small effect sizes, is higher in Acousticness and lower in Speechiness, Energy, Valence, Liveness, Instrumentalness, Danceability and Loudness.

The emergence of Energy and Valence as defining features of our relaxation music dataset is not a surprise. From the Spotify API descriptions, it could be interpreted that these measures relate to the continuous arousal and valence variables of the Circumplex model (Russell, 1980) and changes in either dimension have been found to promote relaxation (Eerola et al., 2024). Regarding our findings on relaxation music being lower in Energy, it is consistent with those observed by Baltazar and Västfjäll (2019), despite differences in comparison groups (i.e., relaxing vs. non-relaxing music in their study and relaxation vs. general music in ours). As with the low Valence of our relaxation music dataset, previous research has also found that people are attracted to using sad music for relaxation purposes (Eerola et al., 2016) and other related self-regulatory functions (Garrido & Schubert, 2011; van den Tol, 2016; van den Tol & Edwards, 2015).

Despite previous studies (e.g., de Witte et al., 2022; Elliott et al., 2011; Wolfe et al., 2002) identifying Tempo as a strong predictor of relaxation music, we found no statistically significant differences in comparing relaxation music to general music. The lack of difference may be due to the variability of relaxation music in our dataset (on which we elaborate on in the next section), or to the method Spotify uses to measure Tempo (e.g., by taking a different metric subdivision like double-time). Though there are some shortfalls to interpreting the Spotify API audio features, we found that the methodology allowed for easy quantitative comparisons which also promoted research with less bias as compared to subjective self-report ratings of perceived relaxation in music used commonly in older studies.

### Variability and clusters of relaxation music

Our second research question pertained to variability in self-selected relaxation music. Analyses revealed some diversity, with three distinct clusters of relaxation music: ‘loud-energetic-danceable’, ‘neutral-acoustic’ and ‘mellow-instrumental’. Previous research by Wolfe et al. (2002) also found that individuals relax to different kinds of music (e.g., classical, soundtrack, country, rock and jazz). Though genre labels can help distinguish the range of relaxation music, Elliott et al. (2011) emphasised the need to move beyond such ‘superficial criteria’ and instead, provide more descriptive details by characterising the music by its musical components (p. 275). We turned to Spotify API audio features to identify and characterise clusters of relaxation music in this study.

The largest cluster in our relaxation music dataset was the ‘loud-energetic-danceable’ cluster which was characterised by low Acousticness and high Loudness, Energy and Danceability. Notably, this cluster had higher mean measures on the audio features it was named after than the general music dataset. The second largest cluster was the ‘neutral-acoustic’ cluster which had less Energy and more Acousticness in comparison. Although the ‘mellow-instrumental’ cluster best reflected commercially labelled and expert-rated relaxation music (i.e., music with high Acousticness and low Valence and Energy), it was the smallest cluster in our dataset. One possible explanation for the size of these clusters might be that our dataset consisted of self-selected music which could have been influenced by individuals’ musical preferences. Previous studies found that ‘pop’ music with moderate tempo and lyrics was preferred for stress recovery (Adiasto et al., 2023), and music with similar characteristics to the ‘loud-energetic-danceable’ cluster has been preferred for pain management (Howlin & Rooney, 2021). The bias towards music from the ‘loud-energetic-danceable’ cluster may additionally be due to our participants, who were mostly young adults–a population which often prefers mainstream music (Woody et al., 2021). In all, this suggests that when people choose music for relaxation, they do not deviate far from their general music listening preferences but rather choose music that is similar to their music choices in everyday life.

### Influences of depression and anxiety scores on relaxation music selection

A significant contribution of this study was the modelling of how individual differences in mental health and musical emotion regulation predicted the selection of relaxation music from different clusters. Regarding mental health, our results showed influences of depression on selecting ‘loud-energetic-danceable’ music and anxiety on ‘mellow-instrumental’ music for relaxation. These findings are in line with prior research which observed that depression and anxiety can shape one’s musical preference and use of strategies for mood regulation (Garrido & Schubert, 2015; Miranda & Claes, 2009; Morgan & Marroquín, 2024; Saarikallio et al., 2015; ter Bogt et al., 2017).

For depressed individuals, the symptoms of depressed mood, low energy and disengagement with interest and pleasure may transfer to a preference for mood-matching music or ‘problem music’ (Garrido & Schubert, 2015; McFerran & Saarikallio, 2014; Paykel, 2008). Consequently, the music may facilitate maladaptive regulation strategies (e.g., rumination and avoidant coping) which reinforce negative states (Garrido & Schubert, 2015; Miranda & Claes, 2009). In other cases, it has been found that depressed individuals may also use ‘upbeat’ music that conveys positivity for adaptive regulation strategies (e.g., mood enhancement, distraction and social connection) that get them out of their felt state (Garrido et al., 2022, p. 9; Saarikallio et al., 2015). The current results revealed that higher depression (HADS-D) scores increased the likelihood of selecting music from the ‘loud-energetic-danceable’ cluster over the remaining two clusters. Based on this finding, it may be suggested that for the purpose of relaxation, individuals with higher scores of depression are more likely to select music that adaptively elevates energy and counteracts negative mood, instead of music that complements their experienced state.

As for anxiety, the clinical psychology literature has acknowledged that there are both adaptive and maladaptive cognitive emotion regulation strategies to help manage symptoms of anxiety (Garnefski & Kraaij, 2018; Schäfer et al., 2017); however, less is known about how these strategies translate to musical emotion regulation and relaxation. Anxiety is often associated with symptoms of tension, hyperactivity and worry (Taipale et al., 2024); thus, music that is arousing and overstimulating may potentially lead to harmful affective outcomes such as maintaining or sustaining stress. Indeed, in exploring everyday cases of successful anxiety self-management through music, Taipale et al. (2024) found that individuals usually had down-regulation goals pertaining to relaxation and calming and used music that was slow and peaceful to achieve such outcomes. Similarly, we found that higher anxiety scores (HADS-A) were related to an increased likelihood of selecting music from the ‘mellow-instrumental’ cluster over the ‘loud-energetic-danceable’ cluster. These findings support past research that has found slow sedative music to be more effective at lowering tension and facilitating relaxation than stimulating music (Iwanaga et al., 1996; Lingham & Theorell, 2009; Sandstrom & Russo, 2010). Together, our findings suggest that individuals intuitively select the appropriate music to achieve relaxation. When listening for this purpose, it may be that individuals do so with the intention to facilitate healthy regulation strategies that suit the needs of their mental state.

### Influences of musical emotion regulation on relaxation music selection

Recent literature suggests that there are several strategies to facilitate relaxation through music (Eerola et al., 2024; Saarikallio et al., 2017). As detailed above, individuals may be selecting different types of music to facilitate these strategies. Our B-MMR model further enforces the idea that individuals are capable of selecting appropriate music for the different regulation strategies.

We showed that increases in the use of Revival predicted a higher likelihood of selecting music from the ‘loud-energetic-danceable’ cluster over the ‘mellow-instrumental’ cluster. Since Revival involves using music to relax or gain energy when stressed or tired, our findings are consistent with past literature that found energetic and groovy music (i.e., music that motivates movement) to be associated with increases in arousal and valence (Cook et al., 2019; Witek et al., 2015).

With regards to the usage of Diversion, our model revealed an increased likelihood of selecting music from the ‘loud-energetic-danceable’ cluster over the ‘neutral-acoustic’ cluster. Since Diversion is a strategy focused on using music to remove unwanted feelings and thoughts, it seems appropriate that energetic music provides enough mental stimulation to redirect individuals’ attention from thinking about their stressors to the musical content.

Lastly, our B-MMR model indicated that increases in the use of Mental Work (i.e., using music to guide contemplation and clarification for emotions and context) and Entertainment (i.e., using music to create or sustain a positive atmosphere) predicted the selection of music from the ‘mellow-instrumental’ cluster over the ‘loud-energetic-danceable’ cluster. Again, this aligns with past literature which has found instrumental music to often be used for focus or background purposes (Scarratt et al., 2023b; Shih et al., 2012).

In general, our findings also reflect the work of Tan & Harrison (2025) who found that music with arousing characteristics was preferred for facilitating B-MMR strategies relating to positive emotions and energy modulation while instrumental music was preferred for strategies that require cognitive reframing and processing. Interestingly, the reported B-MMR strategies showing significant predictions for music selection from a particular cluster are all strategies that are deemed to be adaptive and are healthy ways of using music to regulate mood (Morgan & Marroquín, 2024).

### Limitations and future directions

The current study provides novel results regarding relaxation music and how individual differences relate to its selection; however, it is not without limitations. First, our dataset comprises self-selected music for the context of a relaxation experiment involving a stress induction task (Taipale et al., 2025); therefore, the music in our dataset might not be representative of all everyday situations where musical relaxation may occur. Second, our research focused on individual differences in mental health and musical emotion regulation on music selection for relaxation. Other possible participant background variables (e.g., sensitivity to musical reward, musical sophistication, empathy; Tan & Harrison, 2025; Vuoskoski et al., 2012) which have been found to also influence music selection were not considered. Third, it is important to note that while we interpreted the models of relaxation music selection based on deriving what regulation strategies might best be facilitated by different music and how they may be adaptive to individuals with varying mental health profiles, we cannot confirm the regulation strategies participants had in mind when they provided their music selections.

Future research could consider utilising experience sampling methodology (e.g. Randall & Rickard, 2017), to study more ecologically valid scenarios of musical relaxation while also considering a broader range of music, individual differences and regulation strategies involved (e.g., Randall et al., 2023). To ensure results are more generalisable, future research could also aim to recruit a larger sample of participants ranging in age and culture than those of the current study. One could also take a deeper look into the specific reasons for choosing the relaxation music, or test how effective the music, and perhaps music from a specific cluster, is in terms of facilitating relaxation as measured by self-reports or physiological measures. These questions were excluded from the scope of the current study, but we plan to address them in our future work (Taipale et al., 2025).

## Summary

In conclusion, the study presented here showcases the breadth of self-selected relaxation music and how its selection relates to individual differences in mental health and uses of music for emotion regulation. While no causal explanations can be determined for why the music selection trends exist, we suggest that it may be due to individuals’ intuitive ability to select both adaptive music mood regulation strategies and music to support relaxation. Future research on the efficacy of musical relaxation through these tracks and how it can be translated to naturalistic contexts are still needed; however, the research here raises awareness on the potential of music as a tool for stress alleviation and relaxation while highlighting the importance of considering musical and individual differences. We hope the research advocates and primes the development of accessible music interventions that help promote the fostering of healthier and happier communities.

## Notes

1. It is worth stressing that in the context of Multinomial Logit models, changes in odds between clusters indicate relative likelihoods rather than absolute probabilities. For instance, a rise in the odds of selecting musical tracks from Cluster 1 in relation to Cluster 2 does not suggest that the probability of Cluster 1 surpasses that of Cluster 2; it merely signifies that Cluster 1 has become more likely in comparison to Cluster 2. While probabilities are bounded between 0 and 1 and must sum up to 1 across all clusters, odds, on the other hand, are unbounded and represent ratios of probabilities.

2. The mean VIF value for Model 1 was 1.62. In Model 2, VIF was 2.04 (*SD* = 0.53), ranging between 1.42 and 2.71. In Model 3, VIF was 1.9 (*SD* = 0.85), ranging from 1.10 to 2.74.

3. The chance level here is defined as the proportion of the most represented cluster (i.e., Cluster 1) in the ground truth distribution, which is 54%.
